# Two genotypes of H3N2 swine influenza viruses identified in pigs from Shandong Province, China

**DOI:** 10.3389/fcimb.2024.1517023

**Published:** 2024-12-16

**Authors:** Yuzhong Zhao, Lebin Han, Haotian Sang, Pingping Yang, Yanmeng Hou, Yihong Xiao

**Affiliations:** ^1^ College of Animal Science and Veterinary Medicine, Shandong Agricultural University, Tai’an, China; ^2^ Shandong Provincial Key Laboratory of Animal Biotechnology and Disease Control and Prevention, Shandong Agricultural University, Tai’an, China

**Keywords:** swine influenza virus, H3N2, genetic characterization, mutation, gene

## Abstract

Swine influenza virus (SIV) is a highly contagious pathogen that poses significant economic challenges to the swine industry and carries zoonotic potential, underscoring the need for vigilant surveillance. In this study, we performed a comprehensive genetic and molecular analysis of H3N2 SIV isolates obtained from 372 swine samples collected in Shandong Province, China. Phylogenetic analysis revealed two distinct genotypes. The surface genes of both genotypes clustered with the human-like H3N2 lineage, while the internal genes of one genotype clustered with the 2009 pandemic H1N1 (pdm/09) lineage. In the second genotype, the NS gene clustered with the classical swine (CS) H1N1 lineage, while the remaining internal genes clustered with pdm/09, suggesting stable integration of pdm/09 gene segments into H3N2 SIV. Homology analysis showed over 96% genetic similarity between the isolates and reference strains from China and Brazil, suggesting potential transmission through swine trade or human movement. Molecular characterization identified amino acid substitutions in the HA protein (190D, 226I, and 228S), potentially enhancing the virus’s affinity for human-like receptors, thereby increasing the zoonotic risk. Key mutations in the PB2 (271A, 591R), PA (336M, 356R, 409N), and M2 (S31N) proteins, along with novel drug resistance mutations, indicate the potential for enhanced virulence and drug resistance in these isolates. Moreover, glycosylation site analysis revealed four differences, and antigenic site analysis showed 13 differences between the HA proteins of the isolates and the WHO-recommended vaccine strain A/Cambodia/E0826360/2020 for the 2021–2022 season, which may reduce vaccine efficacy. Serological analysis revealed that 11 out of the tested serum samples were positive for H3N2 antibodies, resulting in an overall positivity rate of 0.42%. These findings emphasize the urgent need for strengthened SIV surveillance in China to monitor the risk of human transmission and ensure better preparedness for future influenza outbreaks.

## 1 Introduction

Swine influenza virus (SIV), a negative-sense, single-stranded RNA virus belonging to the genus Influenza A viruses (IAV) within the family Orthomyxoviridae, is a major pathogen responsible for significant respiratory diseases in swine, leading to substantial economic losses in the global livestock industry. Clinically, SIV infections present as fever, acute bronchitis with respiratory distress, lethargy, and weight loss (Bahari et al., 2015). Pigs play a crucial role in the ecology of IAV, as they are susceptible to both human and avian influenza viruses. This unique susceptibility allows pigs to serve as “mixing vessels,” facilitating the reassortment of influenza viruses and the potential emergence of novel zoonotic strains, as observed during the 2009 pandemic influenza A H1N1 (pdm/09 H1N1) (Vincent et al., 2014; Webster et al., 1992; Chang et al., 2010; Chiapponi et al., 2021).

Currently, multiple subtypes of IAV have been identified in swine populations, but only three—H1N1, H3N2, and H1N2—have been responsible for widespread outbreaks globally (Anderson et al., 2021). The H3N2 IAV first entered swine populations in the 1970s, following the 1968 Hong Kong influenza pandemic A H3N2, marking the earliest recorded case of H3N2 infection in pigs (Shortridge et al., 1977). Since its introduction, H3N2 has spread extensively within swine herds worldwide (Castrucci et al., 1993; Zhou et al., 1999; Tũmová et al., 1976; Shortridge et al., 1979). Notably, in 2011, a variant of H3N2 (H3N2v) emerged in the U.S. swine population, demonstrating the ability to infect humans. This variant carried the matrix (M) gene from the pdm/09 H1N1 virus (Cauchemez et al., 2013; Schicker et al., 2016), highlighting the pivotal role pigs play in generating novel reassortant influenza viruses with zoonotic potential.

In this study, we conducted a detailed genetic and molecular characterization of H3N2 SIV isolates. The findings aim to provide a foundation for improving prevention and control strategies for SIV, contributing to the reduction of cross-species transmission risks and enhancing public health preparedness.

## 2 Materials and methods

### 2.1 Sample collection and processing

A total of 372 samples were collected from nasal swabs, lungs, and trachea of pigs in Shandong Province, specifically from the regions of Linyi, Tai’an, Weifang, Rizhao, Dezhou, and Heze, between November 28, 2017, and March 16, 2023. The samples were immediately placed in a preservation solution consisting of 10% glycerol, 90% phosphate-buffered saline (PBS), 2000 U/ml penicillin, and 2000 U/ml streptomycin. They were kept on ice packs during transport to the laboratory, where they were subsequently stored at -80°C. Lung tissues from H3N2 SIV-positive pigs were collected and fixed in 10% neutral buffered formalin for histopathological examination.

From October 2021 to December 2023, a total of 2,600 serum samples were collected from various pig farms across eight regions in Shandong Province, including Linyi, Tai’an, Weifang, Rizhao, Dezhou, Yantai, Jining, and Heze. All sampled pigs were unvaccinated against any SIV subtypes. Blood samples were drawn from the anterior vena cava using a syringe, and the collected serum was transported to the laboratory under cold conditions. Upon arrival, the samples were left at room temperature for 2 hours before centrifugation at 5,000 × g for 5 minutes to separate the serum. The separated serum samples were stored at -20°C until they were tested for SIV antibodies using the hemagglutination inhibition (HI) assay. Specific antibodies against the H3N2 subtype of SIV were determined using a standard HI test protocol. The H3N2 antigens used for the assay were provided by the National Influenza Reference Laboratory at the Harbin Veterinary Research Institute, Chinese Academy of Agricultural Sciences.

### 2.2 Virus isolation and identification

All samples were inoculated individually into 9–11-day-old specific-pathogen-free (SPF) embryonated chicken eggs for virus isolation. The RNA was reverse-transcribed into cDNA by influenza universal primer Uni12 (5′-AGCAAAAGCAGG-3′) using the ReverTra Ace qPCR RT Kit (TOYOBO, Japan). PCR amplification to detect the presence of SIV was performed using NP gene-specific primers. Samples positive for SIV by PCR were inoculated into the allantoic sac of 9-11-day-old SPF embryonated chicken eggs that were then incubated at 37°C for 48 h before being chilled at 4°C overnight. The allantoic fluid was harvested, filter sterilized, tested for haemagglutination and then stored at −80°C until use.

### 2.3 DNA sequencing

PCR amplification of the viral genomes was performed using gene-specific primers. The PCR products were analyzed using 1% agarose gel electrophoresis and visualized under UV light. DNA sequencing of the PCR products was conducted by Sangon Biotech (Shanghai) Co., Ltd.

### 2.4 Analysis of viral sequencing

The sequencing data were edited using Lasergene Software SeqMan (DNAStar, Madison, WI, USA). Reference virus sequences were retrieved from the Influenza Virus Resource at the National Center for Biotechnology Information (NCBI) database and the Global Initiative on Sharing All Influenza Data (GISAID) database. Amino acid sequence alignment and homology analysis were performed using Lasergene Software MegAlign (DNAStar, Madison, WI, USA). A phylogenetic tree was constructed using the Neighbor-Joining method in MEGA 7.0 software with 1,000 bootstrap replicates. Potential glycosylation sites were predicted using the NetNGlyc 1.0 server (http://www.cbs.dtu.dk/services/NetNGlyc/).

### 2.5 HI tests

The HI test was conducted according to the method described in GB/T 27535-2011 (General Administration of Quality Supervision, Inspection, and Quarantine of the People’s Republic of China, Standardization Administration. Detection method of hemagglutination inhibition antibody against swine influenza [S]. Beijing: Standards Press of China, 2012). Serum samples were treated with trypsin, heat, and potassium periodate to remove nonspecific hemagglutinating inhibitors. Treated serum was then mixed with virus containing four hemagglutinin units, followed by the addition of 1% chicken erythrocytes. HI antibody titers ≥1:10 were considered positive.

## 3 Results

### 3.1 Virus isolation

Between November 28, 2021, and March 16, 2022, a total of 372 swine clinical samples were collected from 32 swine farms across Shandong, China. The samples were sourced from Linyi (n = 103), Tai’an (n = 132), Weifang (n = 25), Rizhao (n = 20), Dezhou (n = 37), and Heze (n = 55). Of these, 14 samples tested positive for SIV by PCR amplification targeting the NP gene (**Table 1**). The positive samples were inoculated into 9- to 11-day-old SPF embryonated chicken eggs and subjected to blind passage for 2 to 3 generations to facilitate virus isolation. In total, 14 samples were isolated and the whole genomes of 6 isolates were sequenced, and the nucleotide sequences were deposited in GenBank (the accession numbers appear in **Table 1**). The HA titer of the harvested chicken embryo allantoic fluid exceeded 2^6^ for all samples (**Table 1**), which were subsequently stored at -80°C for future use.

**Table 1:** Detailed information of samples collected for SIV isolates.

Strain name	Subtype	Date	Cities	HA titer	Clinical Signs	Accession no.
A/swine/Shandong/061/2017	H3N2	12/2017	Heze	2^6^	NO	PQ590145-PQ590152
A/swine/Shandong/0130/2018	H3N2	02/2018	Weifang	2^7^	NO	PQ590052-PQ590059
A/swine/Shandong/0135/2018	H3N2	02/2018	Weifang	2^7^	NO	PQ590134-PQ590141
A/swine/Shandong/0334/2023	H3N2	01/2023	Linyi	2^8^	NO	PQ590064-PQ590071
A/swine/Shandong/0427/2023	H3N2	02/2023	Taian	2^8^	coughing, sneezing	PQ590081-PQ590088
A/swine/Shandong/0438/2023	H3N2	03/2023	Taian	2^9^	coughing, sneezing	PQ590073-PQ590080

NO: No obvious symptoms.

### 3.2 Histopathological changes

**Figure 1:**
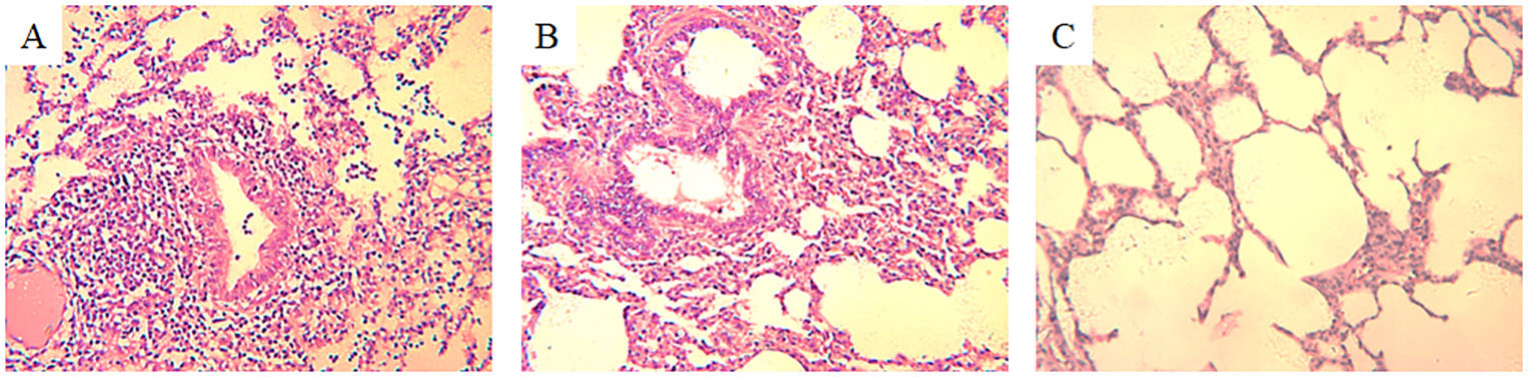
Histopathological changes in the lung tissue of a pig infected with H3N2 SIV. **(A)** Inflammatory cell infiltration, including neutrophils and lymphocytes, can be seen around the bronchioles, with cellular debris partially obstructing the bronchiolar lumen (HE×200). **(B)** Interstitial inflammation and alveolar septal thickening with significant inflammatory cell infiltration and edema in the interstitial spaces of the alveolar region (HE×200). **(C)** Normal lung tissue from an uninfected control pig, showing clear alveolar spaces and intact alveolar septa without signs of inflammation or cellular infiltration (HE×200).

Histopathological examination of H3N2 SIV-positive pig lung tissue showed significant lung lesions. Extensive peribronchial and perivascular infiltrates of monocytes were observed, indicating a strong inflammatory response. In addition, thickening of the alveolar septum and partial filling of the alveolar space by exudate suggest interstitial pneumonia (**Figure 1A**). Bronchial epithelial damage is evident, as well as an infiltration of inflammatory cells in the bronchioles and surrounding area (**Figure 1B**). In contrast, the lung tissue of an uninfected control pig exhibited normal histological features, including intact alveolar structures, thin alveolar septa, and a lack of inflammatory cell infiltration (**Figure 1C**). These findings highlight the pathogenic effects of H3N2 SIV on the respiratory system, leading to severe pulmonary dysfunction and associated clinical symptoms in infected pigs.

### 3.3 Homology analysis

NCBI Blast analysis of the nucleic acid sequences of six H3N2 SIVs revealed that these isolates had more than 96% genetic homology to the closest reference strain in the database (**Table 2**), indicating that they were closely related to the existing reference strains. In addition, the results of the analysis showed significant similarities between these isolates and reference strains from one region of the Porto Alegre country, as well as from several regions of China. The origin of these reference strains reflects the broad geographic distribution of the H3N2 SIV strain. Although these strains come from different geographic regions, the high degree of genetic consistency at the genetic level suggests that there may be a common evolutionary origin or transmission pathway on a global scale.

**Table 2:** The influenza viruses in NCBI with highest nucleotide homology with 6 H3N2 SIV isolates.

AIV gene	Isolate^1)^	Virus sequences with the highest genetic similarity	Homology (%)
PB2	1,2,3	A/swine/Shandong/15/2018(H3N2)	99.8
	4,	A/swine/Shandong/LY142/2017(H1N1)	98.1
	5	A/swine/Liaoning/JZ164/2020(H1N1)	98.7
	6	A/swine/Jiangsu/HD11/2020(H1N1)	98.8
PB1	1,2,3	A/swine/Shandong/15/2018(H3N2)	99.7-99.8
	4,5	A/swine/Liaoning/HLD1795/2020(H1N1)	97.3-97.8
	6	A/swine/Liaoning/TL5239/2020(H1N1)	98.3
PA	1,2,3	A/swine/Shandong/15/2018(H3N2)	99.7
	4,5,6	A/swine/Liaoning/AS1732/2020(H1N1)	98.7
HA	1,2,3	A/swine/Shandong/15/2018(H3N2)	99.1-99.2
	4,5,6	A/swine/Guangxi/2518/2011(H3N2)	96.1-96.2
NP	1,2,3	A/swine/Shandong/15/2018(H3N2)	99.5-99.7
	4,5,6	A/swine/Liaoning/JZ164/2020(H1N1)	98.7
NA	1,2,3,4,5,6	A/swine/Shandong/15/2018(H3N2)	96.3-99.6
M	1,2,3	A/swine/Shandong/15/2018(H3N2)	99.7-99.9
	4,5,6	A/swine/Tai’an/95/2017(H1N1)	98.4
NS	1,2,3	A/Porto Alegre/LACENRS-1771/2009(H1N1)	99.6
	4,5,6	A/swine/Guangxi/1874/2012(H3N2)	97.9

^1)^The H3N2 isolates 1 to 6 were SW/SD/061/17, SW/SD/0130/18, SW/SD/0135/18, SW/SD/0334/23, SWSD/0427/23, and SWSD/0428/23, respectively.

Overall, these data suggest that H3N2 SIV maintains high genetic homology among strains in different geographic regions, supporting its global epidemic and transmission. These findings provide strong molecular evidence for subsequent studies of the evolutionary patterns of H3N2 SIV and its transmission pathways in different regions.

### 3.4 Phylogenetic analysis

**Figure 2:**
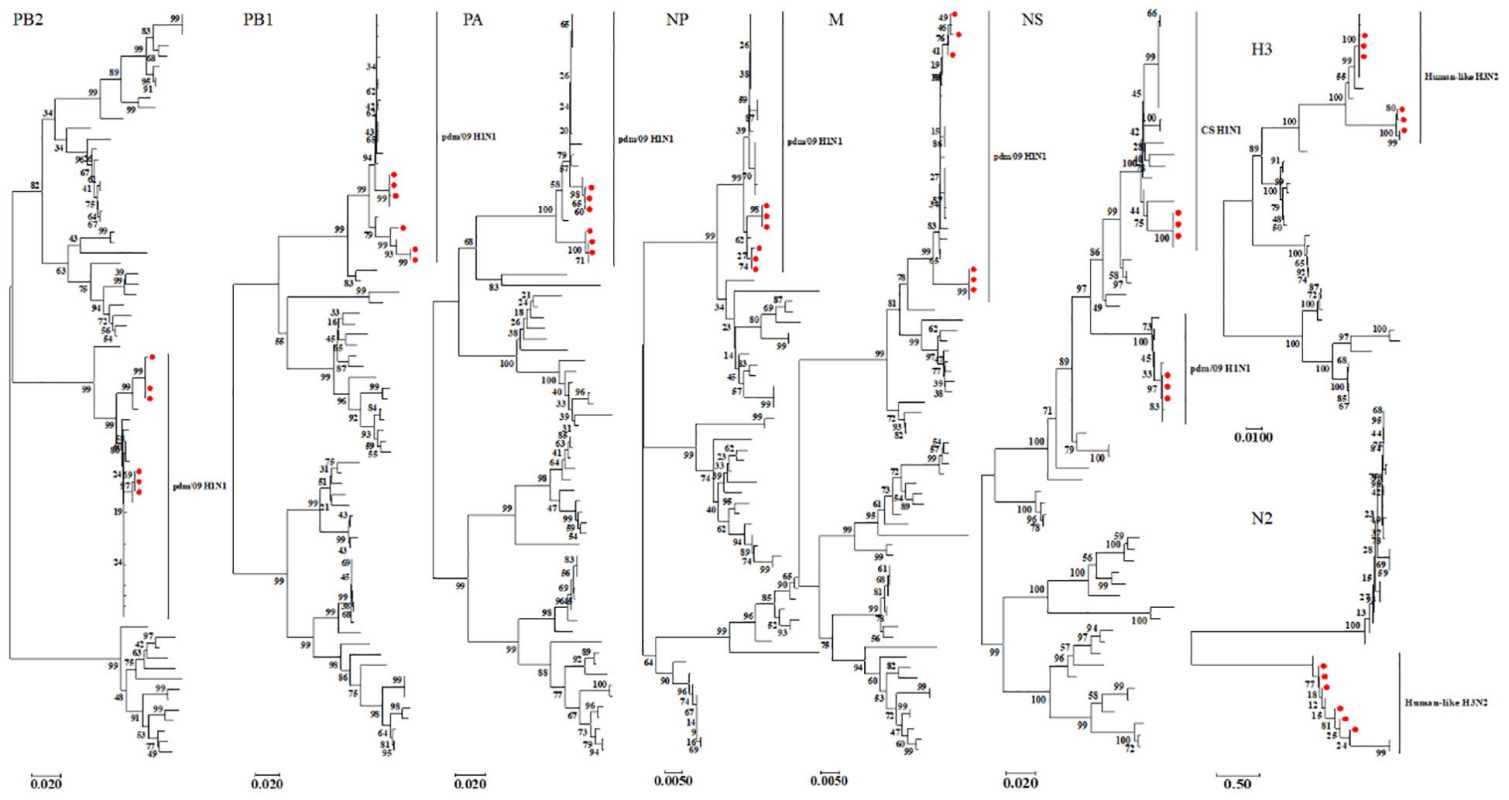
Phylogenetic analysis of the eight genes of the isolated viruses was conducted using the neighbor-joining method in MEGA 7. The reliability of each tree node was assessed through bootstrap analysis with 1,000 replicates. The viruses isolated in this study are indicated by solid red circles.

We constructed eight phylogenetic trees to analyze the evolutionary relationships of the six isolates. For the HA and NA genes, the nucleotide homology ranged from 96.0-99.9% and 94.9-100%, respectively, classifying these two surface genes into the H3N2 Human-like lineage (**Figure 2**). In the case of the internal genes PB2, PB1, PA, NP, M, and NS, the nucleotide homology among the six isolates ranged from 96.2-100%, 95.7-100%, 95.3-100%, 97.5-100%, 97.3-100% and 90.7-100%, respectively. Notably, aside from the NS genes of isolates SW/SD/0334/23, SW/SD/0427/23, and SW/SD/0428/23, which clustered into the CS H1N1 lineage, all other internal genes of the isolates were classified into the pdm/09 H1N1 lineage (**Figure 2**). In addition, we organized the genotypes of H3N2 swine influenza viruses (SIV) in Shandong Province and identified a total of four distinct genotypes (**Table 3**). Since 2005, the evolution of H3N2 SIV isolates over the subsequent years has revealed a complex genetic landscape influenced by various lineages. The earliest isolate, A/swine/Shandong/3/2005 (retrieved from the NCBI database), was identified as a triple-reassortant virus, with its PB2 gene derived from human-like H1N1, the NS gene from classical swine H1N1, and the remaining genes sourced from human-like H3N2 virus. In 2007, a second genotype was identified, including A/swine/Shandong/133/2007 and A/swine/Shandong/106/2007 (both retrieved from the NCBI database), characterized by PB2 and M genes originating from the human-like H1N1 lineage, while all other genes were derived from Eurasian avian sources. In 2017, we recognized a third genotype, represented by SW/SD/061/17, whose surface genes were sourced from the human-like H3N2 lineage, with internal genes stemming from the pdm/09 H1N1 lineage; this genotype was also isolated in 2018, including A/swine/Shandong/15/2018 (retrieved from the NCBI database). In 2023, we discovered a fourth genotype (**Table 3**), including SW/SD/0334/23, SW/SD/0427/23, and SW/SD/0428/23, which all possessed surface genes from the human-like H3N2 lineage, while the internal genes, except for the NS gene belonging to the classical swine H1N1 lineage, were derived from the pdm/09 H1N1 lineage. These results indicate that the internal genes of these isolates have shifted towards the pdm/09 H1N1 lineage, highlighting ongoing gene reassortment events and further emphasizing the adaptive potential of this virus.

**Table 3:** Genotypes of H3N2 SIV in Shandong Province, China from 2005 to 2023.

Strain name	Genotype	Lineage assigned to gene segments							
		PB2	PB1	PA	HA	NP	NA	M	NS
A/swine/Shandong/3/2005	1								
A/swine/Shandong/133/2007	2								
A/swine/Shandong/106/2007									
A/swine/Shandong/15/2018	3								
A/swine/Shandong/061/2017									
A/swine/Shandong/0130/2018									
A/swine/Shandong/0135/2018									
A/swine/Shandong/0334/2023	4								
A/swine/Shandong/0427/2023									
A/swine/Shandong/0438/2023									

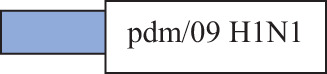
 
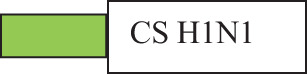
 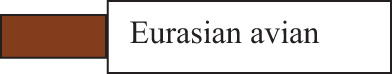

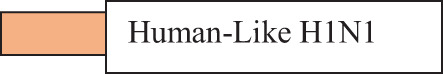
 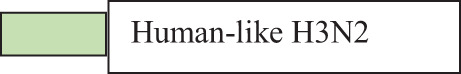

### 3.5 Molecular features analysis

Molecular characterization of the six isolated influenza viruses revealed a cleavage site between HA1 and HA2 with the motif PEKQTR↓GIF, with no insertion of basic amino acids (**Table 4**), indicating a low pathogenicity profile. Previous studies have identified amino acid residues at positions 190D, 226I, and 228S in the HA protein as key determinants for specific binding to the human influenza virus receptor SAα-2,6-Gal (He et al., 2018). In this study, isolates SW/SD/061/17, SW/SD/0130/18, and SW/SD/0135/18 possessed the 226I and 228S residues, while SW/SD/0334/23, SWSD/0427/23, and SWSD/0428/23 exhibited 190D, 226I, and 228S, suggesting a stronger affinity for the SAα-2,6-Gal receptor (**Table 4**).

**Table 4:** Molecular characteristics of isolated viruses.

Gene segment	Amino acids mutations	Biological functions	isolated viruses
HA	190D	Specific for human influenza virus receptor SAα-2,6-Gal binding	190N(3);190D(3)
	226I		226I(6)
	228S		228S(6)
		Cleavage site	PEKQTR↓GIF(6)
PB2	271A	Increased transmission in ferrets	271A(6)
	591R	Increased adaptation in mammalian	591R(6)
	E627K	Increased virulence in mice	627E(6)
	D701N	Increased virulence in mice and transmission in mammals	701D(6)
PB1	I368V	Increased transmission in ferrets	368T(6)
PA	336M	Increased virulence in mice	336M(6)
	356R		356R(6)
	409N		409N(6)
NA	E119V	Drug resistance	119E(6)
	R152K		152V(6)
	R292K		292C(6)
NP	Q357K	Enhanced polymerase activity, replication and infectivity of the virus in mice	357K(6)
M1	N30D	Increased virulence in mice	30S(6)
	T215A		215A(6)
M2	L26F	Drug resistance	26L(6)
	V27A		27I(6)
	A30T/V		30A(6)
	S31N		31N(6)
	G34E		34G(6)
	L38F		38L(6)
NS	P42S	Increased resistance to IFN	42S(6)
	D92E		92D(6)

For the H1N1/2009 pandemic virus, the PB2 271A mutation has been shown to significantly enhance the transmissibility of the virus in ferrets, while the 591R residue is critical for horses (He et al., 2018). All six isolates contained both 271A and 591R mutations in the PB2 protein (**Table 4**). However, no mutations were detected at positions 627 and 701 (**Table 4**), which are known to enhance replication, virulence, and transmission in mammals (Mehle and Doudna, 2008; Tusche et al., 2012).

Glycosylation sites play a crucial role in the antigenicity and receptor-binding characteristics of the HA protein, and they are significant in the emergence of novel viruses (Schulze, 1997). The HA protein of the WHO-recommended vaccine strain E0826360/2020 for the 2021-2022 season contains 10 potential glycosylation sites, while the six isolates in this study contained six potential glycosylation sites at positions 22-NGT, 63-NCT, 126-NWT, 246-NST, 285-NGS, and 483-NGT. The H3 subtype HA protein includes five antigenic sites, designated A, B, C, D, and E. Compared to the WHO-recommended strain A/Cambodia/E0826360/2020 (H3N2), the isolates showed 13 amino acid differences (**Table 5**).

**Table 5:** Antigenic sites of isolated viruses.

Antigenic sites		Viruses	
		E0826360/2020	isolated viruses
A	133-137	NGTSS	NGKSA (4)/NGKSV (1)/NGGSV (1)
	140-146	IRGSSSS	KRGSGNS (3)/KRGSGNS (3)
B	156-160	HLNYK	HLNYK (6)
	187-198	TDKDQISLFAQP	TYDVQISLYTQA (1)/TYNDQISLYTQA (5)
C	51-54	EVTN	EVTN (6)
	276-280	KCKSE	KCNSE (6)
D	174	F	F (6)
	207	K	K (6)
E	63	N	N (6)
	71	L	L (6)
	81	N	N (6)
	83	E	K (6)

The PB1 protein mutation I368V has been shown to enhance viral transmissibility in ferrets (Guo et al., 2020). In contrast, the isolates in this study exhibited a 368T mutation in the PB1 protein (**Table 4**). Moreover, the PA protein mutations 336M, 356R, and 409N, which are associated with increased virulence in mice (Zhang et al., 2023), were present in all the isolates. The NP protein mutation Q356K, known to enhance polymerase activity, replication, and infectivity in mice (Zhao et al., 2023), was also identified in the isolated viruses (**Table 4**).

No mutations were detected at positions E119V, R152V, or R292G in the NA protein, indicating that the isolates remain susceptible to neuraminidase inhibitors (Zhang et al., 2023). The M1 protein mutations N30D and T215A have been linked to increased viral virulence in mice (Guo et al., 2020), and the isolates in this study carried the 215A mutation in the M1 protein (**Table 4**). Amino acid substitutions at positions L26F, V27A, A30T/V, S31N, G34E, and L38F in the M2 protein transmembrane domain have been associated with resistance to adamantane drugs (Ives et al., 2002; 22. Hussain et al., 2017). The isolates carried the S31N mutation, suggesting resistance to adamantanes. Additionally, a novel resistance-associated mutation at position 27 (I) was observed, pointing to continued viral evolution (**Table 4**).

The pathogenicity of influenza viruses in humans is closely related to their ability to evade host antiviral responses, including interferon signaling. Mutations P42S and D92E in the NS1 protein have been shown to enhance viral resistance to interferon (Gu et al., 2013). In this study, the isolates carried the 42S mutation in the NS1 protein, indicating a potential increase in interferon resistance (**Table 4**).

### 3.6 Antibody detection

In the analysis of 2,600 serum samples, only 10 samples tested positive for antibodies against the H3N2 SIV, yielding an overall positivity rate of 0.59%. The number of positive samples recorded from 2021 to 2023 was 2, 4, and 5, respectively, corresponding to annual average positivity rates of 0.23% in 2021, 0.45% in 2022, and 0.58% in 2023 (**Table 6**). This trend suggests a gradual increase in the seroprevalence of H3N2 SIV in the studied regions.

**Table 6:** Serological detection of H3N2 SIV in pig farms in different regions of Shandong Province in 2021-2023.

City	2021			2022			2023			Total		
	P	n	R/%	P	n	R/%	P	n	R/%	P	n	R/%
Linyi	0	150	0.0%	1	150	0.67%	1	100	1.0%	2	400	0.50%
Tai’an	1	127	0.79%	0	106	0.0%	0	170	0.0%	1	403	0.25%
Weifang	0	94	0.0%	0	107	0.0%	1	59	1.69%	1	260	0.38%
Rizhao	0	60	0.0%	0	110	0.0%	0	150	0.0%	0	320	0.0%
Dezhou	1	150	0.67%	1	100	2.0%	1	100	1.0%	4	350	1.14%
Yantai	0	100	0.0%	1	100	1.0%	0	100	0.0%	1	300	0.33%
Jining	0	86	0.0%	1	100	1.0%	1	132	0.76%	2	318	0.63%
Heze	0	88	0.0%	0	114	0.0%	1	100	1.0%	1	302	0.33%
Total	2	855	0.23%	4	887	0.45%	5	859	0.58%	11	2600	0.42%

P. Positive samples; n. Sample number; R. Positive rate.

Among the serum samples collected from large-scale pig farms across eight cities, seven cities reported the presence of H3N2 SIV infection, with antibody positivity rates ranging from 0.25% to 1.14% (**Table 6**). Notably, Dezhou consistently demonstrated the highest positivity rate, indicating potential regional variations in exposure and prevalence of H3N2 SIV among pig farms in Shandong Province.

These findings emphasize the necessity for continued surveillance and monitoring of H3N2 SIV in swine populations, as well as the need for targeted interventions to mitigate the spread of the virus in affected areas.

## 4 Discussion

The pdm/09 virus emerged from a reassortant H1N1 virus with genes originating from multiple sources: PB2 and PA from North American avian viruses, PB1 from human H3N2, HA (H1), NP, and NS from classical swine (CS) H1N1, and NA (N1) and M from Eurasian avian-like H1N1 (EA) (Tam et al., 2013; dos Reis et al., 2011). Following its outbreak in humans, pdm/09 rapidly spread worldwide, causing the first influenza pandemic of the 21st century (Neumann et al., 2009). Subsequently, pdm/09 crossed species barriers, infecting pigs, dogs, cats, and wild mammals (Ma et al., 2015). Currently, pdm/09 is circulating within pig populations globally, reassorting with endemic swine influenza virus (SIV) lineages, leading to a significant increase in reassortant viruses. In China, pdm/09-derived internal genes are frequently isolated from reassortant viruses, suggesting that these genes may be replacing EA and TR internal genes in swine populations (Liang et al., 2014; He et al., 2018; Zhao et al., 2020). In 2011, a novel reassortant H3N2 influenza virus (H3N2v) containing the pdm/09 M gene segment was detected in humans in the U.S (Jhung et al., 2013; Bowman et al., 2014). Limited human-to-human transmission has also been documented (Jhung et al., 2013). Studies suggest that influenza viruses carrying the pdm/09 M gene have a higher likelihood of infecting humans compared to other SIVs (Fan et al., 2012). H3N2v, which carries surface genes from human seasonal H3N2 and internal genes from pdm/09 H1N1, poses a greater risk of rapid, efficient, and sustained transmission in humans (Fan et al., 2012). In 2010, the first recombinant H3N2 SIV carrying pdm/09 internal gene segments was isolated in China (Fan et al., 2012). Since then, multiple reports have emerged regarding recombinant H3N2 SIV strains carrying pdm/09 internal genes (Liang et al., 2014; He et al., 2018; Zhao et al., 2020). Phylogenetic analysis in this study revealed two distinct genotypes among the isolated viruses. The external genes clustered with human H3N2 lineages, while all internal genes of one genotype clustered with the pdm/09 lineage, suggesting zoonotic potential. The second genotype’s NS gene clustered with the CS H1N1 lineage, while the remaining five internal genes clustered with pdm/09. This indicates that pdm/09 internal gene segments have been stably integrated into the H3N2 SIV. The isolation of viruses containing both human and swine influenza virus components reaffirms the role of pigs as “mixing vessels” for reassortment, generating new influenza virus genotypes. Pandemic influenza may arise through two primary mechanisms: the reassortment of animal and human influenza viruses to create new strains, or direct transmission of viruses from animals, followed by adaptation in humans (Yang et al., 2015). In 2019, a case of human infection with an H3N2 SIV was reported in China (Lu et al., 2019). The virus was isolated from a nasopharyngeal swab of a 10-year-old girl and contained surface genes from the HL-H3N2 lineage, with internal genes from pdm/09 H1N1 (PB2, PB1, PA, and NP). The transmission of SIV to humans raises concerns about potential adaptation and subsequent human-to-human transmission, underscoring the need for enhanced surveillance of H3N2 SIV. Pigs play a crucial role in the ecology of IAVs, as they are susceptible to both avian and human influenza strains. Therefore, pigs are considered an intermediate host for interspecies transmission of IAVs or as a “mixing vessel” for the generation of reassortant viruses (Chang et al., 2010). In China, multiple SIV lineages, including CS H1N1, EA H1N1, North American TR, human H1N1/H3N2, and the 2009 pandemic H1N1 (pdm/09), have been established in pig populations (Zhao et al., 2023). Additionally, subtypes like H5N1 and H9N2 have been detected in swine populations, contributing to the diversity of the SIV gene pool, potentially increasing the risk of a pandemic influenza virus emerging.

NCBI Blast analysis indicated that the NS gene of the isolated virus shared the highest homology (99.6%) with a human strain from Brazil, A/Porto Alegre/LACENRS-1771/2009 (H1N1), suggesting that the NS gene may have been introduced into China through human movement or pig trade. The remaining gene segments of the isolated virus showed high homology with corresponding segments from strains in Shandong, Liaoning, and Guangxi provinces, suggesting that these segments may have arisen through reassortment during inter-provincial pig movement.

Molecular analysis revealed that the HA protein of the isolated virus contained amino acid substitutions (190D, 226I, and 228S), which are associated with binding specificity to human SAα-2,6-Gal receptors, indicating potential human infectivity (He et al., 2018). The level of glycosylation in human H3N2 viruses has increased to evade antibody responses, but at the cost of reduced receptor-binding affinity (Liang et al., 2014). The isolated virus differed from the H3N2 vaccine strain (E0826360/2020) by four glycosylation sites, possibly due to reduced antibody selection pressure in pigs. Moreover, the isolated virus exhibited 13 antigenic site differences in the HA protein compared to the H3N2 vaccine strain, suggesting significant antigenic divergence, which may limit the effectiveness of the vaccine against H3N2 SIV. The PA protein (336M, 356R, and 409N) and M1 protein (251A) of the isolated virus may enhance its virulence in mice (Zhang et al., 2023). A Q356K mutation in the NP protein may increase polymerase activity, replication efficiency, and infectivity in mice (Zhao et al., 2023). The S31N mutation in the M2 protein indicates resistance to adamantane antiviral drugs, and a novel resistance mutation at position 27 (I) suggests the virus is continuing to evolve (Ives et al., 2002; Hussain et al., 2017). The NS1 protein carries the 42S mutation, which may enhance the virus’s ability to evade interferon responses (Gu et al., 2013).

The genomic and antigenic characteristics of zoonotic SIV, along with epidemiological data, are essential for assessing the potential risk of these viruses emerging and spreading in human populations. These findings also inform mitigation strategies, such as vaccination programs in swine herds, to reduce the persistent circulation of influenza viruses among pigs. Our study emphasizes the risk of human infection with SIV in China and highlights the need for increased surveillance of both pigs and individuals in close contact with pigs for early detection and characterization of zoonotic influenza infections.

In conclusion, our study provides a comprehensive analysis of the genetic evolution and molecular characteristics of the isolated virus, indicating its potential to infect humans. Continued surveillance of swine populations is crucial for detecting novel influenza viruses that pose threats to both swine and human health.

## Data Availability

The raw data supporting the conclusions of this article will be made available by the authors, without undue reservation.
